# A Multi-Atlas Based Method for Automated Anatomical Rat Brain MRI Segmentation and Extraction of PET Activity

**DOI:** 10.1371/journal.pone.0109113

**Published:** 2014-10-17

**Authors:** Sophie Lancelot, Roxane Roche, Afifa Slimen, Caroline Bouillot, Elise Levigoureux, Jean-Baptiste Langlois, Luc Zimmer, Nicolas Costes

**Affiliations:** 1 Université Claude Bernard Lyon, INSERM, CNRS, Lyon Neuroscience Research Center, Radiopharmaceutical and Neurochemical Biomarkers Team, Lyon, France; 2 Hospices Civils de Lyon, Lyon, France; 3 CERMEP - Imagerie du Vivant, Lyon, France; INSERM U894, Centre de Psychiatrie et Neurosciences, Hopital Sainte-Anne and Université Paris 5, France

## Abstract

**Introduction:**

Preclinical *in vivo* imaging requires precise and reproducible delineation of brain structures. Manual segmentation is time consuming and operator dependent. Automated segmentation as usually performed via single atlas registration fails to account for anatomo-physiological variability. We present, evaluate, and make available a multi-atlas approach for automatically segmenting rat brain MRI and extracting PET activies.

**Methods:**

High-resolution 7T 2DT2 MR images of 12 Sprague-Dawley rat brains were manually segmented into 27-VOI label volumes using detailed protocols. Automated methods were developed with 7/12 atlas datasets, i.e. the MRIs and their associated label volumes. MRIs were registered to a common space, where an MRI template and a maximum probability atlas were created. Three automated methods were tested: 1/registering individual MRIs to the template, and using a single atlas (SA), 2/using the maximum probability atlas (MP), and 3/registering the MRIs from the multi-atlas dataset to an individual MRI, propagating the label volumes and fusing them in individual MRI space (propagation & fusion, PF). Evaluation was performed on the five remaining rats which additionally underwent [^18^F]FDG PET. Automated and manual segmentations were compared for morphometric performance (assessed by comparing volume bias and Dice overlap index) and functional performance (evaluated by comparing extracted PET measures).

**Results:**

Only the SA method showed volume bias. Dice indices were significantly different between methods (PF>MP>SA). PET regional measures were more accurate with multi-atlas methods than with SA method.

**Conclusions:**

Multi-atlas methods outperform SA for automated anatomical brain segmentation and PET measure’s extraction. They perform comparably to manual segmentation for FDG-PET quantification. Multi-atlas methods are suitable for rapid reproducible VOI analyses.

## Introduction

Preclinical neuroimaging studies are increasingly performed on rodents, including rats. Positron emission tomography (PET) imaging in rat animal models, in particular, provides a tool for studying the pathogenesis and progression of neurological disease and for validating new radiotracers and therapeutic agents [1].

Analyzing functional images requires a corresponding anatomical image so as to identify brain structures. Anatomical correspondence is also required to quantify and model interactions between the PET ligand and its pharmacological receptor. Without automated anatomical identification, analyzing PET data relies on time-consuming and observer-dependent manual volume-of-interest (VOI) delineation. Automated delineation usually consists in normalizing images in a reference space, and segmenting the structures in this space using a standardized digital brain atlas. This requires a normalization process, a template (i.e., an averaged image of individuals registered in the reference space, used as target in normalization) and a digital atlas.

MRI templates of rat brain spatially aligned to the Paxinos and Watson anatomical atlas [2] have been previously reported [3], [4], [5]. Digital atlases derived from single subjects have been created from various materials. Digitization and 3D volumetric reconstruction of the Paxinos and Watson atlas co-localized with an MRI template was performed for anatomical and functional interpretation [6], [7]. A digital map of the Sprague-Dawley rat brain, constructed by cryosectioning [8], was used for VOI atlas construction, and was subsequently used in PET studies [9]. High-resolution MR atlases were also developed from fixed rat heads [10], [11] and fixed mouse skulls [12]. Manual delineation of the hippocampus on an individual MRI image was used as an atlas for VOI extraction after affine registration [13]. All these approaches have limitations. They may contain anatomical distortions due to the deformability of frozen brain sections or to ex vivo brain perfusion [14]. They cannot allow for inter-individual anatomical variability, being based on a single image, even if this is obtained by averaging several individual images; moreover, this in itself may lead to inaccurate delineation, as demonstrated in humans. A multi-atlas strategy for segmenting brain structures by propagation and fusion of several brains from a database has been described for the human brain [15], [16], and for Macaca fascicularis [17]. This strategy has proved to be effective, and may be suitable for the rat brain.

The present study introduces two methods for segmenting rat brain MRIs using multi-atlas dataset. This work includes the creation of an MRI template and the multi-atlas dataset. The accuracy of automated segmentations using a multi-atlas approach was tested against single atlas method in a morphological evaluation, and in a functional evaluation using combined MRI and PET data from a test group.

## Materials and Methods

### Animals

Twelve healthy Sprague-Dawley rats were included and underwent MRI. Seven rats (weight 250–350 g) constituted the Atlas group. Five rats (weight 281–330 g) additionally underwent dynamic [^18^F]FDG PET and were used as the Test group in the evaluation procedure. This study was carried out in strict accordance with the recommendations in the guidelines established by the European Communities Council Directive of November 24, 1986 (86/609/EEC). The protocol and the full study were approved by the Committee on the Ethics of Animal Experiments of the University of Lyon (Permit Number: C2EA-42). All imaging sessions were performed under isoflurane anesthesia, and all efforts were made to minimize suffering.

### MRI Acquisition

Animals were scanned in a prone position. To limit the head positioning and slice selection issue, rats were placed on a plexiglas support (Bruker Biospec Animal Handling Systems). The head was maintained in a constrained position with a tooth bar and ear pins, limiting the angulation of the brain and reproducing a transverse plane with limited angulation, in the MRI and in the PET system, since the rat is not moved between the two acquisitions. Breathing rate was monitored throughout the experiment. MRI acquisitions were performed on a 7-Tesla BioSpec System (Bruker BioSpin MRI GmbH, Ettlingen, Germany). T2-weighted contrast images were then acquired axially with a rapid acquisition with relaxation enhancement (RARE) sequence including a fat saturation (FatSat) motif (TR, 8,655.2 ms; TE, 65.3 ms; RARE factor, 8; scan time, 28 min). For 11 rats, 45 slices were acquired with FOV 25.6×25.6 mm, on a 256×256 matrix, resulting in a voxel size of 0.1×0.1×0.5 mm. In 1 rat from the Atlas group, 65 slices were acquired (high resolution rat: HR) with FOV 25.6×25.6 mm and a 256×256 matrix, resulting in resolution of 0.1 mm×0.1×0.4 mm, (TR, 11,063.2 ms; TE, 57.9 ms; RARE factor, 8; scan time, 53 min).

These parameters were chosen to keep the MRI scanning session under 30 minutes. The individual MRIs were manually reoriented with 6 degrees of freedom to align them with the stereotaxic coordinate system of the Paxinos and Watson atlas.

### PET Acquisition

The 5 anaesthetized Test group animals were placed in the PET scanner in a prone position immediately after MRI acquisition, using the same support. PET scans were acquired using a Siemens INVEON PET/CT scanner, with nominal in-plane resolution of ∼1.4 mm full-width-at-half-maximum in the center of the FOV [18]. Before PET radiotracer injection, a CT scan was acquired and used to correct for 511 keV photon attenuation. A 40-minute list mode acquisition started 40 minutes after tail vein injection of the [^18^F]FDG radiotracer (mean, 12.1±1.1 MBq; range, 11.4–14.4 MBq). Images were reconstructed with attenuation and scatter correction by a 3D-filtered back-projection algorithm (Hamming filter; cut-off frequency, 0.5 cycles/pixel) and a zoom factor of 2, resulting in a reconstructed volume of 159 128×128-voxel slices, in a 49.7×49.7×126 mm bounding box, with 0.388×0.388×0.796 mm voxel size. Ten-minute static images from 40 to 50 minutes (PET-FDG) were reconstructed.

### Image Registration

Automated registration used the minctracc program [19]. Intra-individual MR-PET registration used a rigid model consisting of 6 linear transformations (translation and rotation in 3 directions), stored in a transformation matrix. Inter-individual registration (MRI-MRI or MRI-PET) used an affine model followed by a non-linear model. The affine model consisted of 9 linear transformations (translation, rotation and scaling) in 3 directions. The non-linear model consisted of an iterative hierarchical process, with 4 progressively finer resolution levels and maximum iteration numbers: i.e., images were blurred consecutively at 1, 0.5, 0.4, and 0.3 mm FWHM, with progressively finer non-linear control-point spacing of 1.6, 0.8, 0.4, and 0.3 mm. The transformations generated from the non-linear part of the model were stored as a deformation field. The transformation matrices and deformation fields could be applied directly to their source image in order to register them to the space of their target image. They could also be inverted and used to register the target image to the space of the source image. The similarity criterion was mutual information for linear registration, and cross-correlation for non-linear registration. Optimization was achieved with the simplex method. Each registration was visually checked.

### MRI Template

**Figure 1:**
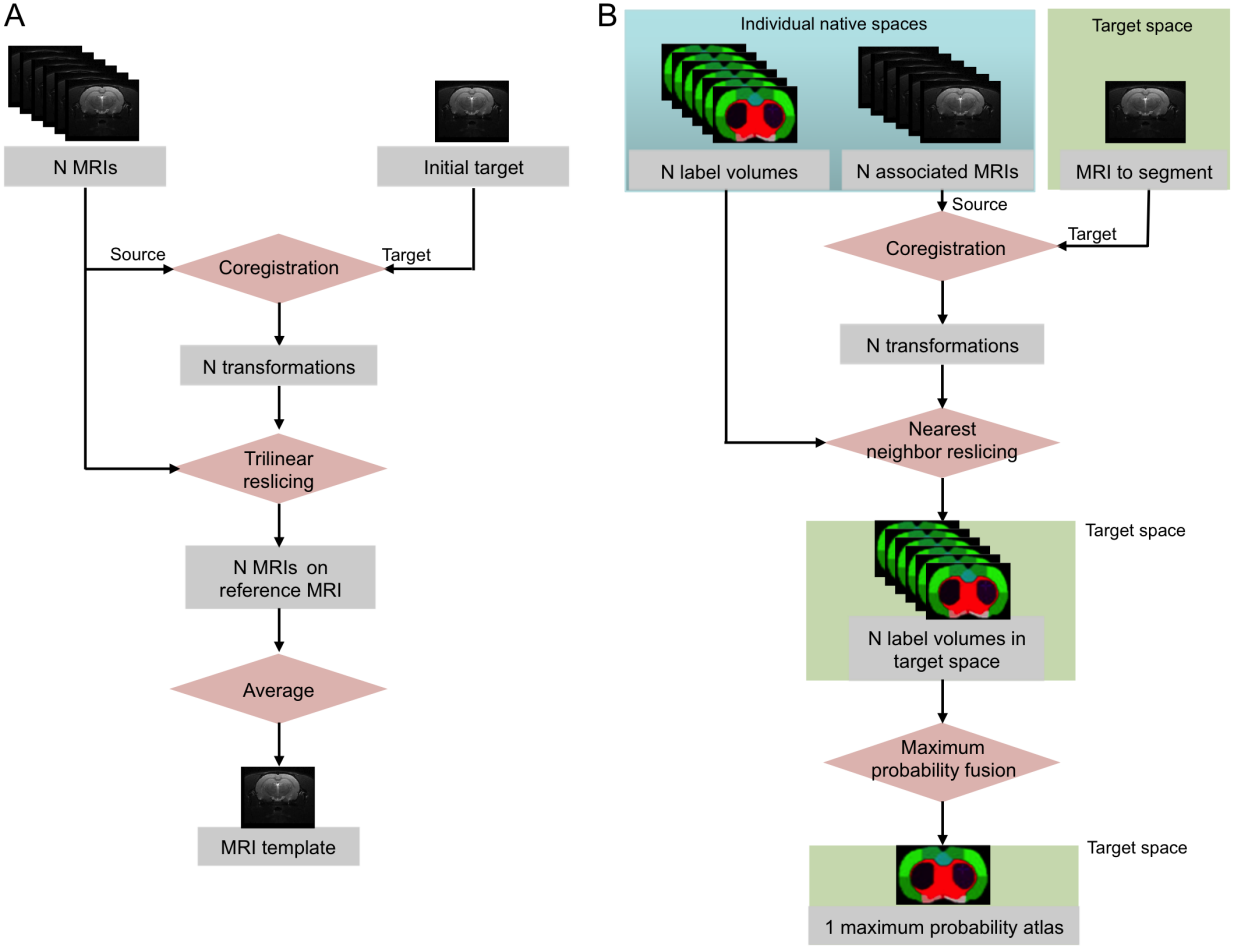
MRI template and maximum probability atlas creation procedures. (A) Creation of the MRI template from N individual MRIs. (B) Creation of a maximum probability atlas from a database of N individual label volumes, associated to the N MRIs.

The MRI template was constructed from images of the 7 Atlas group rats. The template creation procedure is illustrated in Figure 1A. The 65-slice brain MRI was chosen as the initial target. The individual MRIs were manually pre-registered (reoriented, translated and scaled) to match the initial target with, and resliced in cubic voxels. Then, individual MRIs were automatically registered with an affine model on the initial target. The average of the co-registered MRIs constituted the MRI template and defined the template space. The template was sampled in a 256×256×225 matrix with voxel size 0.1×0.1×0.1 mm. The template space was oriented according to the stereotaxic coordinate system of the Paxinos atlas, the first target having previously been oriented manually.

### Multi-Atlas Dataset

#### Creation of the individual atlases

The 7 T2 MRIs in the Atlas group were manually labeled into 27 VOIs, each in native space. The MRI image and its corresponding label volume constitute one atlas of the multi-atlas dataset. The list of these 27 structures, following the nomenclature of the Paxinos atlas, is shown in Table 1. The delineation protocols created for this study can be found in the Appendix S1. VOIs were drawn using *Display* software (MINC, Montreal Neurological Institute, Montreal, Canada). Each volume was primarily drawn on coronal slices, with sagittal and horizontal slices used to confirm regional boundaries. All structures were delineated by one investigator (A.S.) and checked by two others (S.L., N.C.), to ensure protocol conformity.

**Table 1:** Anatomical regions of the digital atlases.

Anatomical regions	Label number
Right caudate + right putamen	1
Left caudate + left putamen	2
Right thalamus + right hypothalamus	19
Left thalamus + left hypothalamus	20
Right hippocampus	21
Left hippocampus	22
Right amygdala	23
Left amygdala	24
Right cerebellum	29
Left cerebellum	30
Right cingulate cortex	33
Left cingulate cortex	34
Right frontal cortex	39
Left frontal cortex	40
Right temporal cortex	43
Left temporal cortex	44
Right occipital cortex	49
Left occipital cortex	50
Right parietal cortex	51
Left parietal cortex	52
Right cortical white matter	80
Left cortical white matter	81
Brain stem	84
Right lateral ventricle	85
Left lateral ventricle	86
Third and fourth ventricles	87
Cerebellar white matter	88

#### Creation of the maximum probability atlas

A maximum probability atlas was created by decision fusion of all 7 individual label volumes in the template space (Figure 1B). Individual label volumes were registered to the MRI template space (see previous section) by applying the spatial transformation of their associated MRIs from native space to this template space. At every voxel, the most likely label in the 7 atlases was then selected by a maximum probability rule [15], [20].

### Automated Multi-Atlas Segmentation Methods

Two automated multi-atlas segmentation methods were implemented, one using the maximum probability atlas, (MP method) and the other an *ad-hoc* maximum probability atlas created by propagation-fusion of the individual atlas dataset in the space of the MRI to be segmented (PF method).

**Figure 2:**
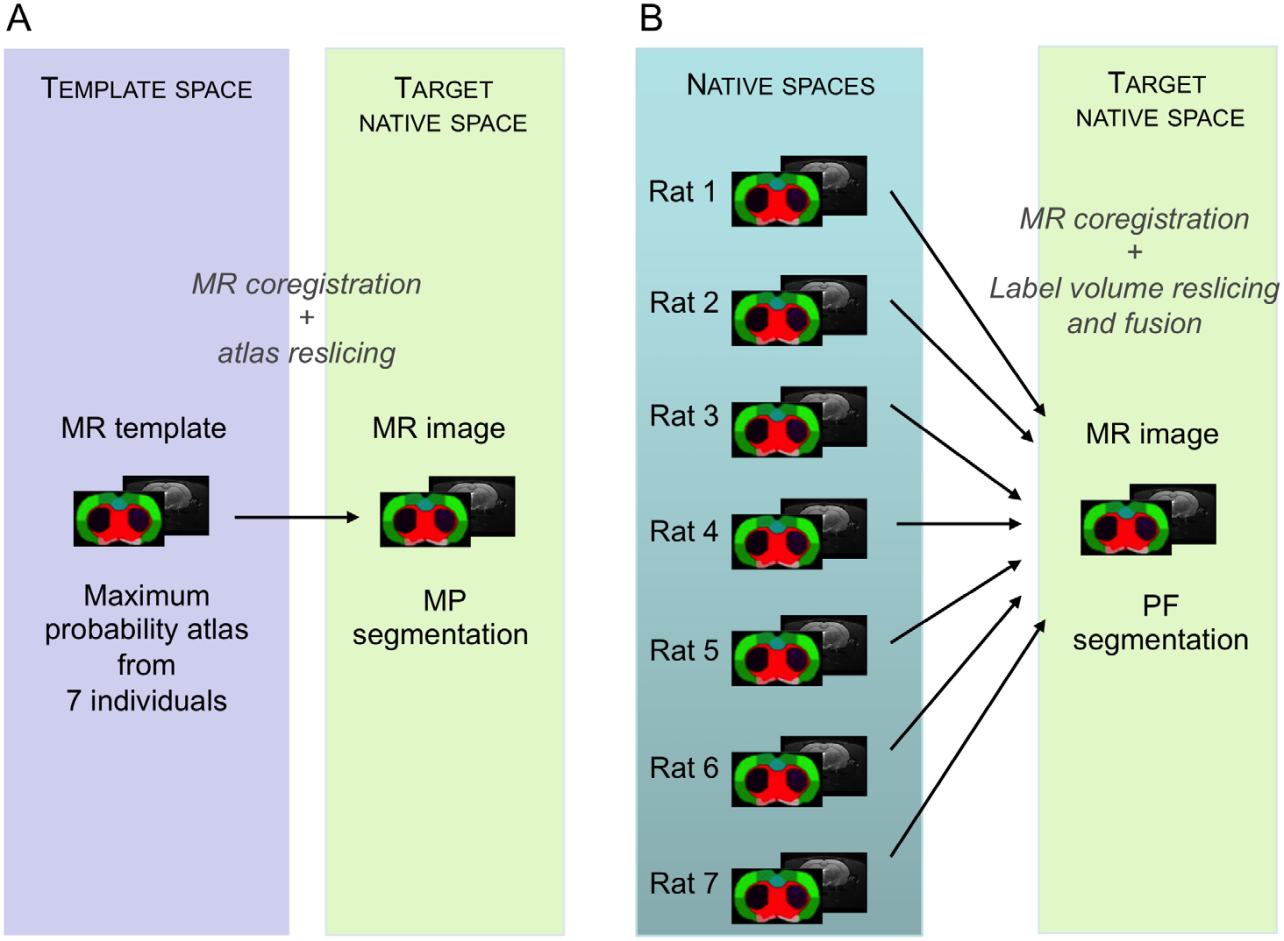
Multi-atlas segmentation methods. MP method (A) using one maximum probability atlas previously created in a template space. PF method (B) using a multi-atlas dataset, and creating an ad-hoc maximum probability atlas in the target space.

The MP method consisted in 1/computing the non-linear registration of an individual MRI on the MRI template; 2/computing the inverse transformation field from template space to individual space; and 3/applying the inverse transformation to the maximum probability atlas and resampling it using the nearest-neighbor (NN) interpolation method to preserve label values (Figure 2A).

The PF method consisted in propagating and fusing the multi-atlas dataset directly in the native MRI space of the subject to be segmented (target), by 1/computing the non-linear registration of each individual MRI from the atlas dataset, onto the target MRI; 2/using the transformations to resample (NN) the label volumes of the atlas dataset in the target space, and 3/performing label fusion of the individual registered label volumes using maximum probability rules (Figure 2B).

### Evaluation

The performance of the multi-atlas segmentation methods was assessed at two levels: *morphometric* and *functional* evaluation.

Evaluation was performed on an independent set of co-registered MRI/PET data: *i.e*., on the five additional rats of the Test group. Brain structures were manually delineated on the individual MRIs with respect to the protocol, this time by different investigators (C.B., S.L., E.L.). All 27 structures were drawn. Accuracy was evaluated in terms of morphometric performance and by measuring functional PET data with automated segmentation.

#### Morphometric evaluation

Three morphometric indices were used to evaluate the automated segmentation methods. To evaluate volumetric performance, relative volume difference (Eq. 1) and the magnitude (absolute value) of the relative volume difference (Eq. 2) were computed, quantifying bias in the structure volume extracted by manual versus automated delineation:
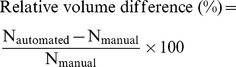
(1)

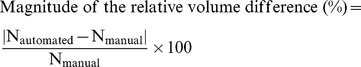
(2)


To evaluate overlap performance, the metric was the Dice similarity index (Eq. 3) [21], which quantifies overlap between manually and automatically delineated structures:
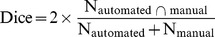
(3)


Dice ranges from 0 (no overlap), to 1 (complete overlap).

N_manual_ is the volume of a manually segmented structure, N_automated_ that of an automatically delineated structure, and N_automated∩manual_ the volume of the overlap between the two.

Morphometric evaluation was performed on the multi-atlas segmentations with the MP and with PF methods. For comparison to more classical segmentation, morphometric evaluation was also performed on regions segmented with an atlas based on a single individual (SA method), instead of the maximum probability atlas in the MP method, using manual delineation of the 65-slice brain MRI.

#### Functional evaluation

Activity extraction was compared between manually delineated and automatically segmented structures with four different methods: with a single atlas (SA), with a maximum probability atlas, when the individual MRI is available (MP with MRI), or without individual MRI (MP without MRI), and with an atlas obtained by a propagation-fusion (PF).

For the MP and SA method, individual PET and MRI acquisitions were registered by computing the rigid spatial transformation of the static PET-FDG image to its MRI. This PET-to-individual-MRI registration was concatenated with the individual MRI-to-template-MRI non-linear transformation. The concatenated transformation was used to transform the single atlas (SA method) or the MP atlas (“MP with MRI” method) in the PET space, where PET-FDG measures were extracted. Alternatively, direct registration between PET-FDG and MRI template was computed. PET-FDG measures were extracted using the resliced MP atlas in the PET space (“MP without MRI”).

For the PF method, propagation-fusion of the multi-atlas dataset was performed with the individual MRI as target, and the *ad hoc* maximum probability atlas was resliced in the PET space to extract regional PET-FDG measures.

Regional [^18^F]FDG activities were converted to standardized uptake values (SUVs). Regression parameters were computed between manual extraction in the individual MRI space versus SA, MP (with or without MRI) and PF methods.

### Statistical Analysis

All statistical analyses were performed using STATA 8 (StataCorpLP, College Station, TX, USA). The significance threshold was set at p<0.05. Morphological and functional performances of the methods were assessed by paired t-tests with reference to the SA method. Functional performance was evaluated with regression analysis with reference to the manual method. P-values were corrected for multiple comparisons with the Bonferroni method.

## Results

**Figure 3:**
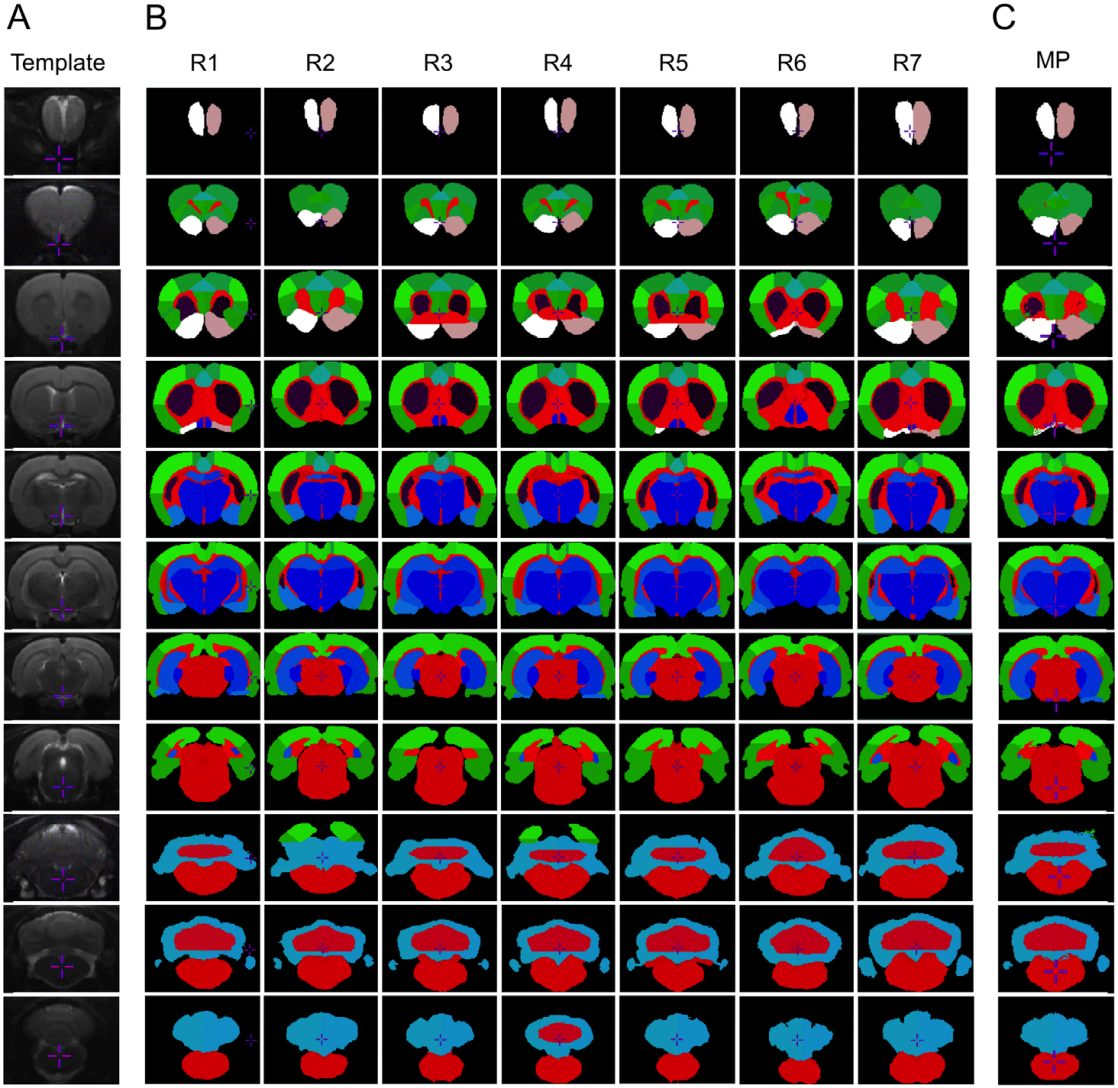
Multi-atlas dataset and maximum probability atlas. Rat MRI template (A) composed from 7 individuals. Dataset of 7 individual atlases (B). Maximum probability atlas (C), combination of the 7 individual atlases. Coronal slices, 2 mm apart (one slice out of five are shown).

The MRI template was created by combining the 7 registered individual MRIs in the template space (Figure 3A). Template creation was repeated, with the first template as target. Since there was no further improvement in superposition accuracy, the first template was considered satisfactory. Seven individual label volumes were drawn (Figure 3B) and the maximum probability atlas combining the 7 individual label volumes were created (Figure 3C).

### Morphometric Evaluation

For information, volumes of the manually delineated regions are given in Table 2 (first column). There was a mean 3% variation in inter-individual volumes over regions. The results of the morphometric evaluation are shown in Table 2.

**Table 2:** Morphometric evaluation.

	Manual			Single Atlas (SA)						MaximumProbability Atlas (MP)									Propagation/Fusion Atlas (PF)								
	Volume (mm^3^)			Volume bias (%)			Dice			Volume bias (%)					Dice				Volume bias (%)					Dice			
Regions	mean		sd	mean		sd	mean		sd	mean			sd		mean			sd	mean			sd		mean			sd
Right caudate + putamen	42.03	±	1.43	6.53	±	2.50	0.851	±	0.012	2.15		±	1.61		0.873	*	±	0.009	2.11	*	±	1.63		0.875	**	±	0.007
Left caudate + putamen	40.67	±	0.27	9.09	±	5.00	0.840	±	0.006	4.78		±	1.95		0.863	*	±	0.012	5.05		±	0.93	**	0.869	***	±	0.007
Right thalamus + hypothalamus	79.70	±	0.99	10.62	±	1.31	0.855	±	0.010	1.60	***	±	1.19		0.901	***	±	0.007	1.60	***	±	1.41		0.901	***	±	0.007
Left thalamus + hypothalamus	78.04	±	1.13	5.16	±	2.90	0.852	±	0.009	2.87		±	2.07		0.897	***	±	0.008	2.48		±	2.33		0.899	***	±	0.007
Right hippocampus	56.86	±	2.10	8.07	±	2.21	0.833	±	0.008	2.67		±	2.62		0.864	***	±	0.005	3.50		±	2.61		0.868	***	±	0.007
Left hippocampus	54.22	±	2.05	12.16	±	3.77	0.835	±	0.009	4.81		±	3.84		0.854		±	0.020	3.56		±	2.73		0.867	***†	±	0.014
Right amygdala	21.34	±	1.04	15.55	±	2.51	0.738	±	0.021	1.53	***	±	1.57		0.792	**	±	0.028	2.07	***	±	0.58	*	0.794	***	±	0.028
Left amygdala	20.71	±	1.01	10.32	±	4.44	0.747	±	0.018	3.40		±	3.33		0.775		±	0.050	2.23		±	3.05		0.778		±	0.050
Right cerebellum	93.59	±	1.40	5.44	±	3.24	0.762	±	0.017	1.62		±	0.59	**	0.806	**	±	0.016	1.69		±	1.27		0.816	***††	±	0.018
Left cerebellum	95.10	±	1.73	14.52	±	3.45	0.770	±	0.018	4.32	**	±	4.00		0.805	*	±	0.024	5.42	**	±	3.16		0.817	**†	±	0.019
Right cingulate cortex	12.22	±	0.34	6.06	±	5.16	0.774	±	0.049	10.64		±	6.90		0.764		±	0.047	14.58		±	9.30		0.746		±	0.054
Left cingulate cortex	12.60	±	1.05	4.88	±	4.22	0.797	±	0.035	8.30		±	6.91		0.769		±	0.037	9.31		±	3.87		0.769		±	0.035
Right frontal cortex	55.05	±	2.64	9.56	±	4.33	0.792	±	0.033	9.20		±	2.35		0.805		±	0.021	8.60		±	3.02		0.800		±	0.032
Left frontal cortex	56.10	±	0.95	8.40	±	3.98	0.792	±	0.019	6.70		±	3.64		0.801		±	0.018	7.88		±	4.36		0.809	*	±	0.017
Right temporal cortex	113.07	±	1.87	2.22	±	1.27	0.800	±	0.008	3.97		±	1.99		0.824	*	±	0.006	2.04	†	±	1.93		0.828	**	±	0.009
Left temporal cortex	114.49	±	4.33	1.95	±	1.75	0.792	±	0.017	2.41		±	1.69		0.811	*	±	0.025	2.77		±	2.26		0.815	**	±	0.018
Right occipital cortex	55.58	±	1.91	8.23	±	6.67	0.822	±	0.026	3.37		±	2.01	*	0.866	*	±	0.010	4.73		±	3.03		0.855		±	0.015
Left occipital cortex	53.69	±	4.43	4.56	±	2.51	0.826	±	0.022	3.63		±	2.78		0.852	**	±	0.028	4.92		±	3.12		0.842		±	0.026
Right parietal cortex	78.22	±	4.56	2.90	±	1.76	0.880	±	0.006	3.71		±	2.56		0.890	*	±	0.005	7.21	*†	±	3.49		0.884		±	0.011
Left parietal cortex	76.64	±	3.57	3.97	±	2.32	0.867	±	0.010	4.25		±	2.15		0.871		±	0.017	5.80		±	3.14		0.877		±	0.008
Right cortical white matter	80.48	±	4.75	2.92	±	2.03	0.687	±	0.010	1.85		±	1.58		0.734	***	±	0.006	1.84		±	1.25		0.739	***	±	0.006
Left cortical white matter	79.24	±	3.28	3.67	±	2.16	0.684	±	0.013	7.50		±	3.31		0.732	**	±	0.020	5.05	††	±	2.84		0.737	***	±	0.016
Brain stem	302.10	±	8.70	8.92	±	3.12	0.887	±	0.012	7.32		±	2.95		0.919	**	±	0.006	6.38		±	3.33		0.920	**	±	0.005
Right lateral ventricle	7.40	±	0.95	13.15	±	10.89	0.557	±	0.042	10.72		±	6.76		0.614	*	±	0.016	9.49		±	6.52		0.625	*	±	0.016
Left lateral ventricle	7.17	±	0.74	6.77	±	6.16	0.573	±	0.042	4.03		±	6.16		0.603		±	0.033	10.23		±	5.92		0.643	*††	±	0.028
Third and fourth ventricles	25.56	±	1.94	21.15	±	4.67	0.601	±	0.040	5.42	**	±	1.81		0.669	**	±	0.020	9.20	***	±	2.54		0.679	**	±	0.021
Cerebellar white matter	81.22	±	0.78	13.004	±	1.996	0.860	±	0.014	7.70	**	±	3.13		0.880	**	±	0.013	2.68	***†	±	1.76		0.885	*	±	0.016
**Whole brain**	**1793.11**	±	**56.65**	**8.14**	±	**5.86**	**0.780**	±	**0.091**	**4.83**	***	±	**4.13**	***	**0.809**	***	±	**0.084**	**5.27**	***	±	**4.55**	**	**0.813**	***†††	±	**0.079**

Volume bias = magnitude of the relative volume difference. Whole brain does not include olfactory bulb. N = 5.

Mean comparison: paired t-test, SA vs MP or PF (*:p<0.05; **:p<0.01; ***:p<0.001), MP vs PF (†:p<0.05; ††:p<0.01; †††:p<0.001).

Sd comparison: F-test, SA vs MP or PF (*:p<0.05; **:p<0.01; ***:p<0.001), MP vs PF (†:p<0.05; ††:p<0.01; †††:p<0.001).

P-values are corrected for multiple comparisons.

The average of the relative volume difference was non-zero for SA (1.388±9.721%; p>0.05) whereas, due to a small standard deviation, it did not differ from zero for MP (−0.996±6.235%; p<0.05) or PF (−1.889±6.588%; p<0.001).

Magnitude of the relative volume differences and Dice indices are given in Table 2. The mean magnitude of the relative volume difference was 8.139±5.860% for SA and lower overall for MP (4.832±4.130%; p<0.001) and PF (5.274±4.555%; p<0.001). With SA, maximum bias in volume exceeded 22% (3^rd^ and 4^th^ ventricles), but was significantly reduced to ∼5% (p<0.001) with MP method and to ∼9% (p<0.001) with PF. The magnitude of the relative volume difference on multi-atlas methods was significantly reduced for 5 structures with MP and for 7 with PF. The standard deviation of the volume difference magnitude (inter-individual variability) was also reduced overall with MP (p<0.001) and PF (p<0.01). The most significant improvements in volume measurement accuracy with multi-atlas methods were in the thalamus/hypothalamus, amygdala, cerebellum, cerebellar white matter and 3^rd^ and 4^th^ ventricles.

The multi-atlas approach significantly increased mean Dice index compared to the single atlas approach.

Overall, Dice index increased from 0.780 with SA to 0.809 with MP (p<0.001) and 0.813 with PF (p<0.001). However slight the increase, PF performed significantly better than MP (p<0.001). This improvement in Dice index was seen in almost all regions (19/27 for MP and 19/27 for PF). Additionally, PF provided better overlap than MP in 4 regions: left hippocampus, right and left cerebellar gray matter, and left lateral ventricle. Inter-individual variability (mean coefficient of variation) in Dice index ranged from 11.6% for SA to 10.4% for MP and 9.7% for PF, although these differences were non-significant.

### Functional Evaluation

**Figure 4:**
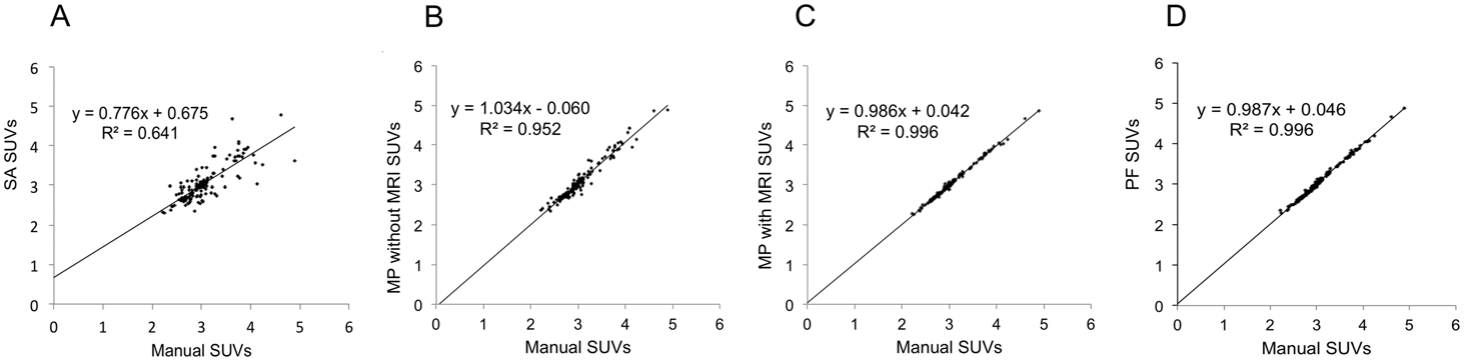
Application for functional measure automated extraction. Regression graphs of [^18^F]FDG regional standard uptake values (SUVs) obtained with automated segmentation methods, compared to SUVs obtained with manual segmentation. (A) SA method, (B) MP method performed with direct registration of the PET image to the template MRI, (C) MP method, (D) PF method.

The accuracy of [^18^F]FDG PET regional measurement (SUVs) with MP and PF was compared with measurement of manually delineated regions. Regressions, shown in Figure 4, showed a good correlation (R^2^ = 0.641; p<0.0001) between single atlas and manual SUVs, and excellent correlations between multi-atlas and manual SUVs (Figure 4. B, C, D). Even the extraction method that did not use individual MRI was excellent (R^2^ = 0.952; p<0.0001,) despite slight (non-significant) scatter (Figure 4B).

Comparison of individual regressions parameters (slope, intercept and coefficient of determination of the regression, Table 3) showed than multi-atlas methods perform better than single atlas method.

**Table 3:** Functional evaluation: regression against extraction with manual atlas.

Method	Slope				Intercept				R2			
	mean			SD	mean			SD	mean			SD
SA	0.428		±	0.144	1.762		±	0.618	0.177		±	0.057
MP with PET	0.933	***	±	0.097	0.240	**	±	0.285	0.861	***	±	0.033
MP with MRI	0.975	***	±	0.031	0.075	***	±	0.090	0.985	***	±	0.009
PF	0.985	***	±	0.029	0.051	***	±	0.084	0.981	***	±	0.014

Mean comparison (N = 5): paired t-test versus SA (*:p<0.05; **:p<0.01; ***:p<0.001).

P-values are corrected for multiple comparisons.

#### Computation time

Typically, segmentation and functional data extraction took 5 minutes on a single central processing unit (AMD Opteron dual core 1 GHz processor running RedHat) with MP and less than 40 minutes with PF. These times are to be compared with the dozens of hours needed for manual delineation.

## Discussion

A multi-atlas dataset and anatomical MRI template of the Sprague-Dawley rat were created. The accuracy of automated segmentation with the multi-atlas dataset was checked. Regional PET measurement extraction with an automated atlas was also shown to be equivalent to extraction by manual VOI delineation.

### Morphometric Evaluation of Multi-Atlas Segmentation

Automated segmentation with a multi-atlas dataset did not induce significant bias in VOI measurement compared to manual delineation. The rat is an animal model widely used for the study of neurological diseases, including degenerative processes that may induce regional volumetric alterations. The present multi-atlas method is an accurate and reproducible means of studying large datasets, since it can be performed automatically in a reasonable computing time. Volumetric measurement was, however, assessed on healthy cases, and the inter-individual variability of the structural database was therefore limited to populations without lesions. Study of pathological cases that may be associated with greater morphological alteration might need more sophisticated non-linear registration methods [22], [23] and, further, methods using diffeomorphic registration and minimal deformation templates [24]. The minimal deformation target (MDT) strategy uses pairwise non-linear image registration to create an average template with minimal deformation, derived from each of the initial brain images [25]. One of the limits of our template creation was its simplicity, and in future work, the use of MDT and unbiased target selection for template creation might improve our method. MRI acquisitions with more isotropic voxels might also benefit the registration method by avoiding interpolation artifacts. In our study, we had chosen to maximize the in-plane resolution coronally to align the structural delineation of brain regions with the Paxinos atlas. In future work, acquisitions with more isotropic voxels in the same field of view could be achieved by reducing the in-plane resolution, which would allow reducing the section thickness while respecting the constraint of maintaining a MRI scan time of less than 30 minutes.

In the Test group, multi-atlas methods achieved overall performance of more than 0.8 for the Dice overlap index. To our knowledge, no previous performance results for a multi-atlas approach in rat have been reported in the literature; studies in bee [26] or human brain [27] using a similar methodology (multi-atlas and maximum probability decision fusion) reported a performance of 0.8 as standard. There are, however, approaches that are closer to the present study: the MAGeT method is a propagation-fusion method for mouse brain segmentation, based on a single model of VOI segmentation, propagated to 25 individual MRIs, with non-linear deformation, then propagated and fused on the subject to be segmented [28]. With a mouse model, the best result for segmentation is obtained in the hippocampus, with a Dice index of 0.869 (95% confidence interval (CI), 0.853–0.884). With the present delineation protocol, the Dice index in the hippocampus was 0.864 (CI, 0.854–0.874) with MP and 0.868 (CI, 0.848–0.882) with PF. For comparison, results obtained with the single-atlas method were significantly poorer, with a mean Dice of 0.833 (CI, 0.817–0.849; p<0.001 compared to multi-atlas methods). We conclude that the present results are within the performance range of other multi-atlas methods, although rigorous comparison should be performed using a single database and single delineation protocol.

Additionally, performance might be increased by more sophisticated fusion rules and atlas selection. The present study finally provided 12 atlases, since the initial multi-atlas dataset of 7 can be completed by the 5 of the Test group. With a dataset of 12 atlases, we would be able to implement a multi-atlas method where decision fusion is not performed with the whole dataset, but by selecting a custom subset of atlases. It has been shown in human studies that selecting atlases with an image-based similarity criterion performs better than a random selection, or than using the whole dataset [29]. These approaches would allow longitudinal study of an evolving brain lesion, or others pathophysiological brain processes in rodents.

### Segmentation for Functional Quantification

PET regional measurements showed excellent agreement between the manual and automated multi-atlas delineation methods. It was firstly checked that activity extracted by individual manual delineation was completely equivalent in the individual PET, individual MRI and template spaces (r^2^≈1, data not shown). Measures were then extracted using multi-atlas automated delineation in the MRI space. Regression slopes for manually extracted data were close to 1; correlation coefficients were very good, with r^2^ = 0.996 (MP and PF), the regression slope was close to 1. It was concluded that, in spite of the imperfect overlap between manually and automatically delineated regions, the multi-atlas methods were sufficiently precise to be able to extract PET measurements accurately. The tolerability of the slight mismatch of regions is due to the lower resolution of PET compared to MRI: the small segmentation error visible with the MRI resolution was negligible at the resolution of the PET acquisitions. Our data also shows that using a single individual to create an atlas is largely sub-optimal in comparison to the use of a dataset of multiple atlases that are combined in a maximum probability atlas. The transformations from the individual space and the template space used in the SA and MP methods are the same. They do not explain differences in terms of performance. The SA is clearly biased because it does not contain the inter-individual variability of the structural delineations of a brain region. The MP atlas performs better because it is created from different individuals, and does contain inter-individual variability. Because the MP atlas has a higher signal to noise ratio as a result of averaging, it might be a better target for automated image registration. In addition, multi-atlas methods may better account for subtle differences in slice orientation between different acquisitions which may be present despite the rats being imaged with head restraints.

The case of automated extraction of rat brain PET data without using a corresponding individual MRI is of particular interest. Because individual MRIs are frequently not available in rat brain PET studies, we tested automated PET data extraction by directly registering the PET image to the MRI template, using inverse transformation of the maximum probability template in the PET space. In this context, the availability of a specific ad-hoc template is a key point and, to this end, future studies of combined MRI/PET acquisition in rats are planned, using various PET tracers. For example, thanks to this work, a static normalized FDG template has been created by averaging individual static FDG normalized in the template space. Their normalization parameters have been initially computed via PET-to-MRI and MRI-to-template-MRI. This average constitutes a specific FDG template, usable for further spatial normalization of PET data without MRI.

## Conclusions

A multi-atlas based method with non-linear transformation for automatically segmenting rat brain MRI and extracting PET activities was created and validated. Multi-atlas methods outperformed the single-atlas method for automated brain segmentation, and also performed comparably to manually defined regions for PET quantification. Additionally to rapid and reproducible VOI analysis, the definition of a template space will be helpful for intra- and inter-individual voxel-based longitudinal multi-tracer analysis. The maximum probability atlas and the individual atlas dataset are available, with grant of license, from CERMEP, University of Lyon.

**Disclosure:** All authors have read and agreed with the contents of the manuscript. The results of the study have not been published before and they are not under consideration to be published by another journal.

## Supporting Information

Appendix S1 Delineation protocol for manual segmentation.
(DOCX)
