# Supplementary material for: A Multi-Atlas Based Method for Automated Anatomical Rat Brain MRI Segmentation and Extraction of PET Activity
Source: PLoS One. 2014 Oct 17;9(10):e109113. doi: 10.1371/journal.pone.0109113 (PMC4201469; doi:10.1371/journal.pone.0109113)
Supplement: Appendix S1 — Delineation protocol for manual segmentation. (DOCX) [file pone.0109113.s001.docx]

**APPENDIX**

**Delineation Protocol**

**APPENDIX: MANUAL DELINEATION**

Structures 1 and 2: caudate-putamen (right; left)

Orientation of slices Coronal

Anterior border First slice: slice anterior to that in which lateral ventricles are clearly visible

Posterior border Last slice: slice anterior to that in which inferior hippocampus horns are visualized (see structures 21 and 22)

Medial border Anterior → posterior: lateral ventricles, cortical white matter (see structures 80 and 81)

Lateral border Anterior → posterior: cortical white matter

Superior border Anterior → posterior: cortical white matter, lateral ventricles

Inferior border Anterior → posterior: cortical white matter, amygdala

Size (mm) ~ 6

Structures 19 and 20: thalamus (right; left)

Including thalamic and hypothalamic nuclei

Orientation of slices Coronal

Anterior border First slice: most anterior slice in which third ventricle is seen

Posterior border Last slice: starting on first slice in which the aqueduct of Sylvius is distinguished

Medial border Anterior → posterior: CSF, brain stem

Lateral border Anterior → posterior: cortical white matter, amygdala, lateral ventricles, hippocampus

Superior border Anterior → posterior: cortical white matter, CSF, hippocampus, brain stem

Inferior border Anterior → posterior: CSF, brain stem, hippocampus, cortical white matter

Size (mm) ~ 6.4

Structures 21 and 22: hippocampus (right; left)

Orientation of slices Coronal

Anterior border First slice: slice in which cingulate cortex disappears and occipital cortex appears

Posterior border Last slice: first slice in which right and left occipital cortices are clearly separated in midline

Medial border Anterior → posterior: cortical white matter, CSF, thalamus, brain stem

Lateral border Anterior → posterior: lateral ventricles, cortical white matter, temporal cortex

Superior border Anterior → posterior: cortical white matter, lateral ventricles

Inferior border Anterior → posterior: CSF, thalamus, amygdala, temporal cortex

Size (mm) ~ 6.6

Structures 23 and 24: amygdala (right; left)

Including the centromedial, basolateral, and cortical nuclei.

Orientation of slices Coronal

Anterior border First slice: defined on the most anterior slice in which the third ventricle is seen

Posterior border Last slice: slice anterior to that in which the aqueduct of Sylvius is visible

Medial border Anterior → posterior: cortical white matter, thalamus, CSF

Lateral border Anterior → posterior: temporal cortex, cortical white matter

Superior border Anterior → posterior: cortical white matter, caudate-putamen, lateral ventricles, hippocampus

Inferior border Anterior → posterior: cortical white matter, CSF

Size (mm) ~ 5.6

Structures 29 and 30: cerebellum (right; left)

Excluding cerebellar white matter

Orientation of slices Sagittal

Medial border Cerebellar white matter

Lateral border CSF

Anterior border Medial → lateral: brain stem, CSF, temporal cortex; coronal cut drawn containing the aqueduct of Sylvius

Posterior border CSF

Superior border Medial → lateral: CSF, brain stem, temporal cortex

Inferior border Medial → lateral: CSF, brain stem

Size (mm) ~ 14.8

Structures 33 and 34: cingulate cortex (right; left)

Orientation of slices Coronal

Anterior border First slice: most anterior slice in which corpus callosum is visualized

Posterior border Last slice: slice anterior to that in which hippocampus appears (as previously defined)

Medial border Anterior → posterior: no medial border

Lateral border Anterior → posterior: frontal cortex, cortical white matter

Superior border Anterior → posterior: frontal cortex, CSF

Inferior border Anterior → posterior: temporal cortex, cortical white matter (corpus callosum)

Size (mm) ~ 6

Structures 39 and 40: frontal cortex (right; left)

Orientation of slices Transverse

Anterior border Superior → inferior: CSF, olfactory bulb

Posterior border Superior → inferior: parietal cortex, temporal cortex, cortical white matter

Medial border Superior → inferior: CSF, cingulate cortex, occipital cortex, cortical white matter, temporal cortex, olfactory bulb

Lateral border Superior → inferior: CSF, parietal cortex, temporal cortex

Superior border CSF

Inferior border Temporal cortex, parietal cortex, cortical white matter, olfactory bulb

Size (mm) ~ 7

Structures 43 and 44: temporal cortex (right; left)

Orientation of slices Coronal

Anterior border First slice: starting on first slice in which frontal cortex is visualized

Posterior border Last slice: slice anterior to that in which cerebellar white matter appears

Medial border Anterior → posterior: frontal cortex, cortical white matter, CSF, amygdala, hippocampus, brain stem

Lateral border Anterior → posterior: frontal cortex, CSF, cortical white matter

Superior border Anterior → posterior: frontal cortex, cingulate cortex, parietal cortex, occipital cortex, CSF

Inferior border Anterior → posterior: frontal cortex, olfactory bulb, CSF

Size (mm) ~ 14.8

Structures 49 and 50: occipital cortex (right; left)

Orientation of slices Coronal

Anterior border First slice: first slice in which hippocampi are distinguished

Posterior border Last slice: last slice in which temporal cortex is seen

Medial border CSF

Lateral border Anterior → posterior: frontal cortex, parietal cortex, CSF

Superior border CSF

Inferior border Anterior → posterior: cortical white matter, brain stem, temporal cortex, CSF

Size (mm) ~ 8

Structures 51 and 52: parietal cortex (right; left)

Orientation of slices Coronal

Anterior border First slice: most anterior slice in which cortical white matter joins olfactory bulb

Posterior border Last slice: beginning of aqueduct of Sylvius

Medial border Anterior → posterior: cortical white matter, frontal cortex, occipital cortex

Lateral border Anterior → posterior: CSF, temporal cortex

Superior border Anterior → posterior: frontal cortex, CSF, occipital cortex

Inferior border Anterior → posterior: temporal cortex, cortical white matter

Size (mm) ~ 8.8

Structures 80 and 81: cortical white matter (right; left)

The area remaining after drawing the other structures

Including basal forebrain, lateral globus pallidus, nucleus accumbens, lateral and medial septum, corpus callosum, external capsule, internal capsule, bed nucleus of the stria terminalis

Structure 84: brain stem

Orientation of slices Coronal

Anterior border First slice: beginning of the aqueduct of Sylvius

Posterior border Last slice: slice posterior to that in which cerebellum disappears

Medial border CSF

Lateral border Anterior → posterior: thalamus, hippocampus, cortical white matter, temporal cortex, occipital cortex, CSF, cerebellum

Superior border Anterior → posterior: CSF, thalamus, hippocampus, occipital cortex, cortical white matter, cerebellum

Inferior border Anterior → posterior: thalamus, CSF, cerebellum

Size (mm) ~ 10.4

Structures 85 and 86: lateral ventricle (right; left)

Orientation of slices Coronal

Anterior border First slice: most anterior slice in which caudate-putamen are clearly identified

Posterior border Last slice: slice anterior to that in which brain stem begins

Medial border Anterior → posterior: cortical white matter, hippocampus, thalamus

Lateral border Anterior → posterior: caudate-putamen, cortical white matter, amygdala

Superior border Anterior → posterior: cortical white matter

Inferior border Anterior → posterior: cortical white matter, thalamus, caudate-putamen, amygdala

Size (mm) ~ 6.8

Structure 87: third and fourth ventricles

Including third and fourth ventricles and CSF

Orientation of slices Coronal

Anterior border First slice: end of olfactory bulb

Posterior border Last slice: slice anterior to that in which cerebellum finishes

Medial border No medial border

Lateral border Cortical white matter, olfactory bulb, thalamus, hippocampus, brain stem, cerebellum

Superior border Cortical white matter, thalamus, hippocampus, brain stem, occipital cortex, cerebellum

Inferior border Thalamus, brain stem

Size (mm) ~ 14.8

Structure 88: cerebellar white matter (right; left)

Including the white matter of the cerebellum in a convex envelope that necessarily also includes gray matter

Convex envelope drawn within the cerebellum, similar in shape to the lateral border of the cerebellum, with the following limits

Orientation of slices Sagittal

Medial border Midline

Lateral border 1.5 mm deep from the lateral cerebellar border

Anterior border 1 mm deep from the anterior cerebellar border

Posterior border 1 mm deep from the posterior cerebellar border

Superior border 1.5 mm deep from the superior cerebellar border

Inferior border 1.5 mm deep from the inferior cerebellar border

Size (mm) ~ 9
